# The Nordic Nutrition Recommendations 2022 – principles and methodologies

**DOI:** 10.29219/fnr.v64.4402

**Published:** 2020-06-18

**Authors:** Jacob Juel Christensen, Erik Kristoffer Arnesen, Rikke Andersen, Hanna Eneroth, Maijaliisa Erkkola, Anne Høyer, Eva Warensjö Lemming, Helle Margrete Meltzer, Þórhallur Ingi Halldórsson, Inga Þórsdóttir, Ursula Schwab, Ellen Trolle, Rune Blomhoff

**Affiliations:** 1 Norwegian National Advisory Unit on Familial Hypercholesterolemia, Oslo University Hospital, Oslo, Norway; 2 Department of Nutrition, University of Oslo, Oslo, Norway; 3 National Food Institute, Technical University of Denmark (DTU), Kgs. Lyngby, Denmark; 4 The Swedish Food Agency, Uppsala, Sweden; 5 Department of Food and Nutrition, University of Helsinki, Helsinki, Finland; 6 The Norwegian Directorate of Health, Oslo, Norway; 7 Department of Environmental Health, Norwegian Institute of Public Health, Oslo, Norway; 8 School of Health Sciences, University of Iceland, Reykjavík, Iceland; 9 Department of Medicine, Endocrinology and Clinical Nutrition, Kuopio University Hospital, Kuopio, Finland, and Institute of Public Health and Clinical Nutrition, University of Eastern Finland, Kuopio Campus, Kuopio, Finland; 10 Division of Cancer Medicine, Oslo University Hospital, Oslo, Norway

**Keywords:** dietary reference values, food-based dietary guidelines, systematic reviews, Nordic countries, the Baltics, national food and health authorities, evidence-based nutrition, nutrient recommendations

## Abstract

**Background:**

The Nordic Nutrition Recommendations (NNRs) constitute the scientific basis for national dietary reference values (DRVs) and food-based dietary guidelines (FBDGs) in the Nordic and Baltic countries.

**Objective:**

To define principles and methodologies for the sixth edition of NNR to be published in 2022 (NNR2022).

**Design:**

The principles and methodologies of the previous edition of NNR were used as a starting point. Recent nutrition recommendations commissioned by other national food and health authorities or international food and health organizations were examined and dissected. Updated principles and methodologies were agreed by the NNR2022 Committee in a consensus-driven process.

**Results:**

An organizational model with ‘checks and balances’ was developed to minimize the influence of subjective biases of the committee members and experts. Individual chapters on all included nutrients and food groups will be updated as scoping reviews. Systematic reviews (SRs), which are the main basis for evaluating causal effects of nutrients or food groups on health outcomes, will be embedded in each chapter. A NNR SR Centre will be established for performing *de novo* SRs on prioritized topics. To avoid duplication and optimize the use of resources, qualified SRs commissioned by other national and international organizations and health authorities will also inform DRVs and FBDGs in NNR2022.

**Discussion:**

The evidence-based methods defined in the NNR2022 project are compatible with most contemporary methods used by leading national food and health authorities. Global harmonization of methodological approaches to nutrition recommendations is strongly encouraged.

**Conclusion:**

Evidence-informed principles and methodologies underpinned by SRs will ensure that DRVs and FBDGs defined in the NNR2022 project are based on the best available evidence and as far as possible free from overt bias.

## Popular scientific summary

- The Nordic and Baltic countries are developing a common scientific basis for national nutrition recommendations.
- The project is commissioned by the Nordic Council of Ministers.
- A comprehensive organizational model with ‘checks and balances’ was developed.
- Contemporary evidence-based principles and methodologies based on the best available evidence were defined.

The Nordic Nutrition Recommendations (NNR) constitute the scientific basis for national dietary reference values (DRVs) and food-based dietary guidelines (FBDGs) in Denmark, Finland, Iceland, Norway, and Sweden, as well as Estonia, Latvia, and Lithuania. The international collaboration has resulted in the previous five editions of the NNR (1). The recommendations are intended for generally healthy populations, considering different age groups, and pregnant and lactating women. Since first published in 1980, the NNR has been regularly updated (1).

This article is the first of a three-part series for the sixth edition of the NNR (NNR2022):

1. *Principles and methodologies* (this article),
2. *Structure and rationale of qualified systematic reviews* (2), and
3. *Handbook for qualified systematic reviews* (3).

Together, these documents constitute a comprehensive and precise framework for how we plan to update the NNR. The present paper, paper one, describes the organization, principles, methods, and the systematic approach used for the upcoming version to be published in 2022 (NNR2022). Herein, we define *a priori* methodology and data handling for clarity and transparency. This is especially important for food and health authorities when defining DRVs and FBDGs. Similarly, the second paper, *Structure and rationale of systematic reviews* (2), describes *a priori* all aspects related to the SR methodology that will be used to evaluate the selected diet-health associations. The third paper, *Handbook for systematic reviews* (3), is a guide on how to conduct SRs according to the structure and rationale outlined in the second paper.

The main objective of NNR2022 is to use the best available scientific evidence to ensure a diet that provides energy, food, and nutrients for optimal growth, development, function, and health throughout life. It should be noted that certain DRVs and FBDGs are only applicable if the supply of other foods and nutrients are adequate.

Historically, the NNR has included the following DRVs for nutrients: 1) average requirement (AR); 2) recommended intake (RI); 3) upper intake level (UL); 4) lower intake level (LI); and 5) reference values for energy. Recently, NNR has also included FBDGs.

The principles and methodologies for developing DRVs and FBDGs have evolved considerably over the last few years. For example, during the last decade, SRs have been established as the preferred method for evaluating causality between diet and health outcomes (4–9). The methods used in SRs for synthesizing the strength of evidence for specific dietary exposures and health outcomes vary, and the methodology is likely to continue to develop. It is especially challenging to balance the emphasis on clinical trials and randomized controlled trials (RCTs) versus observational studies and mechanistic studies in order to evaluate the strength of evidence for a causal association between diet exposures and chronic disease outcomes (see discussion below).

Due to possible further development of the principles and methodology for developing DRVs and FBDGs, some aspects defined in the present paper may be changed at later stages of the project. Any potential deviation will be clearly stated in the final report.

## Sponsors of the NNR2022 project

The following sponsors have supported the work with NNR2022:

- Nordic Council of Ministers
  - Nordisk arbeidsgruppe for kosthold, mat og toksikologi (NKMT), The Nordic Council
  - Samarbeidsministrene(MR-SAM), TheNordic Council
  - Nordisk ministerråd for fiskeri og havbruk, jordbruk, næringsmidler og skogbruk (MR-FJLS), The Nordic Council
  - Nordisk embetsmannskomite for fiskeri og havbruk, jordbruk, nærinmgsmidler og skogbruk (EK-FJLS), The Nordic Council
- Food and health authorities in Denmark, Finland, Sweden, and Norway

The mandate from the sponsors was to update the fifth edition of the NNR (published in 2014) based on new scientific evidence. The only specific request was that sustainability needed to be implemented in NNR2022. The main position/affiliation for HE, EWL, and HMM were their respective national food and health authorities (see *Affiliations*). The Steering Committee (see below) also consists of members from national food and health authorities. Otherwise, the sponsors will have no direct role in the NNR2022 project.

## Organization of the NNR2022 project

**Fig. 1:**
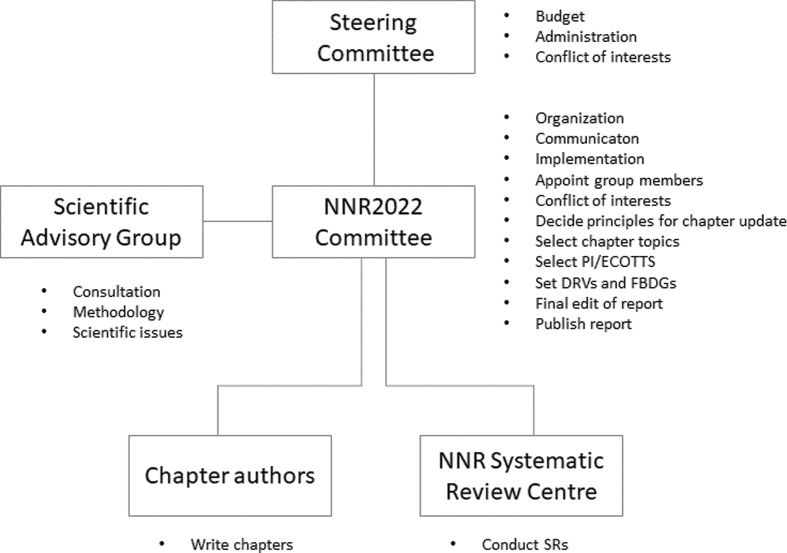
The NNR2022 project: organization and responsibilities.

The NNR2022 project is founded on solid scientific traditions, and this is also the case for the organization of the project. Although we, the NNR2022 Committee members (see next subsection), are the main drivers of the project, we have designed an organizational system of ‘checks and balances’ that tries to minimize the influence of *our* innate or unconscious biases. The NNR2022 project consists of five organizational parts, explained in Fig. 1.

### The NNR2022 Committee

The NNR2022 Committee is responsible for organizing and implementing the NNR2022 project and publishing the final NNR2022 report. The NNR2022 Committee is appointed by the national food and health authorities in Denmark (RA, ET), Finland (ME, US), Iceland (ÞIÞ, IÞ), Norway (JJC, HMM), and Sweden (HE, EWL). Representatives appointed by food and health authorities in Estonia, Latvia, Lithuania, as well as the autonomous areas of the Faroe Islands, Greenland, and the Åland Islands have been welcomed to contribute to the NNR2022 Committee as observers. The committee has 11 members (including the project leader RB, who is from Norway), five observers, and one project secretary (AH).

The NNR2022 Committee is responsible for appointing the Scientific Advisory Group, the NNR Systematic Review Centre, chapter authors, and referees, and for approving any conflict of interest for the Scientific Advisory Group, chapter authors, and peer reviewers. If there are any conflicts of concern, the NNR2022 Committee will consult the Steering Committee.

The NNR2022 Committee is responsible for project progress and coordination, communication with the Steering Committee, organization of relevant workshops, public hearings, and the NNR2022 websites.

Based on initial scoping reviews (ScRs) and public consultations, the NNR2022 Committee will select chapter topics for SRs and decide how to update each individual chapter. The committee is also responsible for the final editing of all chapters and the setting of DRVs and FBDGs.

The NNR2022 Committee will arrange several public hearings, call for experts and topics for SR, and strive to be as transparent as possible in all considerations related to the project.

### The Steering Committee

The Steering Committee consists of five representatives from the food and health authorities in the Nordic countries (Henriette Øien, The Norwegian Directorate of Health, Oslo, Norway; Satu Männistö, Finnish Institute for Health and Welfare, Helsinki, Finland; Hólmfríður Þorgeirsdóttir, Directorate of Health, Reykjavík, Iceland; Ulla-Kaisa Koivisto Hursti, National Food Agency, Uppsala, Sweden; Anne Pøhl/Else Molander, Danish Veterinary and Food Administration, Glostrup, Denmark), and is chaired by the representative from Norway. The responsibilities of the Steering Committee are to approve the budget, set the criteria for conflict of interest, and evaluate the declaration of conflict of interest for the NNR2022 Committee. The Steering Committee will also regularly approve progress reports from the NNR2022 Committee.

### The Scientific Advisory Group

The Scientific Advisory Group is appointed by the NNR2022 Committee after consultation with the Steering Committee. The group should consist of international scientists with experience in developing DRVs and FBDGs for national authorities or health organizations, as well as to have competence in the field of sustainability. As such, they will advise on the principles and methodologies for developing the sixth edition of the NNR, including the methodology used to perform SRs as part of the NNR2022 project. They will also give advice on general scientific issues related to the NNR2022 project.

### The NNR Systematic Review Centre

The NNR2022 project will fund a NNR Systematic Review Centre (NNR SR Centre), which will be responsible for performing all SRs on topics prioritized by the NNR2022 Committee.

The NNR SR Centre will consist of a team of at least six scientists from different disciplines. At least two of the members will be senior scientists and one should be a statistician, all three with experience in conducting SRs. The other members may be PhD students or postdocs. In addition, the NNR SR Centre will be facilitated by two librarians. The NNR SR Centre team will be selected by the NNR2022 Committee based on invitations by the NNR2022 Committee and a public call. Representatives from all the Nordic countries will be included in the NNR SR Centre, if possible.

### Chapter authors

The NNR2022 project will engage scientific experts across the Nordic and Baltic countries to update the chapters. Experts outside these countries may also be engaged. All experts and referees will be listed in the relevant chapter. At least two experts will be assigned to a chapter, one of which will serve as a leading author. The experts will be appointed by the NNR2022 Committee based on invitations by the NNR2022 Committee and a public call, after careful evaluation of each expert´s skills and experience related to the chapters. The NNR2022 Committee members should avoid being authors on the nutrient and food chapters.

### Peer reviewers for chapters and systematic reviews

In addition to the main organization, at least two experts will be appointed to peer-review individual chapters and SRs. These experts will be appointed by the NNR2022 Committee based on direct invitation by the NNR2022 Committee and a public call. Personal skills and competence related to the individual chapters or SR will guide the choice of peer reviewers.

## Conflicts of interest

Almost all scientists have some sort of direct or indirect conflict of interest. Conflict of interest may arise due to the institution where the scientist is employed; many state institutions receive external funding directly or indirectly from commercial interests or interest organizations. Many private or commercial institutions may also have specific interests related to their purpose or ideology.

Conflict of interest may also arise due to funding to the scientist of concern. All scientists must compete for internal and external resources for scientific activities. The external sources that fund most research span from national research funds that distribute resources from governmental budgets, to patient or interest organizations (e.g. cancer-, heart- or diabetes funds) and commercial entities (e.g. pharmaceutical industry and food producers). Furthermore, governmental funds, including those resources distributed through European Union and national research councils, most often demand collaboration with commercial companies. Industry-sponsored research is therefore a large part of modern medical science. Otherwise, the development of newer drugs, and medical technologies for the detection and treatment of disease would not have been possible.

### The organization of the NNR project reduces the risk of individual bias

The objective of the NNR project is to develop updated DRVs and FBDGs, as objectively as possible, without the influence or bias of the scientists involved. That is why we have designed an organizational system of ‘checks and balances’ that minimizes the influence of the innate biases of the scientists involved. Some important features of this system are that:

1. the project is split into discrete parts that will be done by separate experts,
2. the project involves a large number of experts from a large number of nutrition and non-nutrition subdisciplines,
3. we avoid the likelihood of experts influencing multiple parts of the process,
4. peer-review will ensure scientific quality and integrity of the final output, and
5. we strive to be as open and transparent as possible in all matters related to the project.

### Handling of conflict of interest in the NNR2022 project

Due to sponsorship for research from commercial entities and ideological organizations, concerns have been raised about bias in the results of such research. For example, evidence for substantial bias has been identified in conclusions of industry-sponsored systematic reviews. It is suggested that industry-sponsored research will result in approximately 30% higher likelihood off a favorable conclusion, compared to nationally sponsored research (4, 5). While industry-sponsored research is likely to be important for nutrition research also in the future, it is fundamentally important that industry sponsors should have no role in project design, implementation, analysis, or the interpretation of results. This independence greatly minimizes the potential for bias. These issues are carefully evaluated in studies considered by the NNR2022 project.

Furthermore, to reduce the risk of such bias, NNR2022 does not consider SRs commissioned or sponsored by industry or organizations with a business or ideological interest as qualified SR. Only SR commissioned by national food or health authorities, or international food and health organization, may be identified as qualified SR (for definition of qualified SR, see section ‘Identification of qualified Systematic reviews’).

Due to the impact of the NNR2022 project, a number of industry and ideological organizations are expected to contact the NNR 2022 Committee during the project period. In order to prevent any influence of such contacts on scientific conclusions, the NNR2022 project only accepts inputs from such sources through the official website of the NNR project. All inputs through this website will be fully open and available for everybody. General contact with industry and ideological organizations is expected in open symposia or workshops.

### Declaration of interests

The central goal of the conflict of interest policies is to protect the integrity of professional judgment and to preserve public trust. The disclosure of individual and institutional financial relationships is a critical step in the process of identifying and responding to conflict of interest. NNR2022 experts and committee members will declare, on a specific form, all relationships from which they (and their spouse or dependent children) received either assets or supplemental income of greater than 10.000 DKK in the last 5 years outside of compensation related to their full-time, permanent employment.

Experts with strong ties to industry or ideological organizations are also excluded from serving as chapter authors, peer reviewers, NNR SR Centre members, or other committee members. Thus, relevant industry or ideological ties will also be declared.

## Procedure for updating chapters in NNR2022

Chapters in NNR2022 will follow a standardized format, outlined in an *Instruction to authors* guide. The main chapter subsections are listed in Table 1, together with the type of review on which they will be based. Generally, all chapters can be regarded as ScRs. Some chapters will contain one or several qualified SRs, while others will contain none. Importantly, different types of reviews and studies will feed into the different subsections in each chapter. Due to the lack of availability of qualified SRs, no one chapter will be fully based on qualified SRs.

**Table 1:** Different types of reviews feed into different subsections in each NNR2022 chapter

Chapter subsection	Basis
Physiology	Scoping review
Determination of nutrition status	Scoping review
Exposures relevant for Nordic and Baltic countries	Scoping review
Health outcomes relevant for Nordic and Baltic countries	Scoping review
- Total mortality	*de novo* systemic review, qualified systemic review, or scoping review
- Obesity	*de novo* systemic review, qualified systemic review, or scoping review
- Cardiovascular diseases	*de novo* systemic review, qualified systemic review, or scoping review
- Cancers	*de novo* systemic review, qualified systemic review, or scoping review
- Diabetes type 2	*de novo* systematic review, qualified systemic review, or scoping review
- Osteoporosis	*de novo* systemic review, qualified systemic review, or scoping review
- Other health-related outcomes	*de novo* systemic review, qualified systemic review, or scoping review
- Hypertension	*de novo* systemic review, qualified systemic review, or scoping review
- Dyslipidemia	*de novo* systemic review, qualified systemic review, or scoping review
- Dysglycemia	*de novo* systemic review, qualified systemic review, or scoping review
Requirements and recommended intakes	Scoping review
Integration (physical activity, obesity, and sustainability)	Scoping review

### Systematic reviews are the preferred method to evaluate causality

Since around 2010, national health authorities and international organizations have gradually started to use SRs as the preferred method for evidence-based evaluation of causal relations between nutrient or food exposures and health outcomes. SRs follow a strict *a priori* defined procedure and conclude with a grading of the evidence for a causal association (also called ‘strength of evidence’ and ‘quality of evidence’). The EQUATOR network has formulated requirements that must be met in reporting SRs (6).

NNR should ideally build on updated SRs of highest quality for *all* associations between nutrient/food groups and health-related outcomes. However, due to the high cost and resources involved in SR research, this is not feasible. Therefore, for certain associations, we will identify and include recently conducted qualified SRs (see ‘Identification of qualified Systematic reviews’ section that can be used in the evaluation of causal associations). In addition, for a limited selection of other associations, the NNR Systematic Review Centre will perform *de novo* SRs (see ‘Process for selecting topics for systematic reviews’ section).

### Scoping reviews of topics not covered by systematic reviews

For subsections of the NNR chapters not covered by *de novo* SRs or other qualified SRs, the text will follow the procedures of the PRISMA Extension for Scoping Reviews (PRISMA-ScR) defined by the EQUATOR Network (Table 1) (7). A ScR is a more transparent method to develop and report scientific reviews than the narrative or classic literature reviews often used when setting DRVs or FBDGs (Box 1).

## Process for selecting topics for systematic reviews

**Fig. 2:**
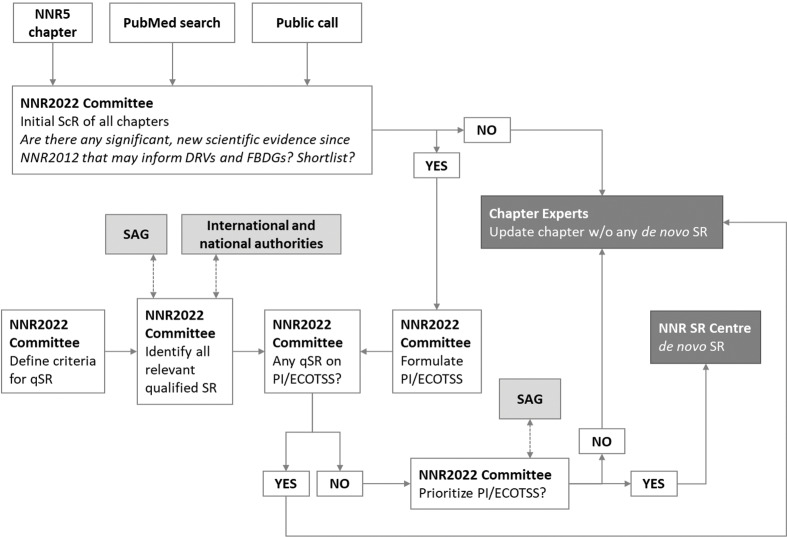
The process for prioritizing and selecting topics for SRs. Abbreviations: SAG, Scientific Advisory Group; qSR, qualified systematic review; ScR, scoping review; NNR SR Centre, NNR Systematic Review Centre; DRVs, dietary reference values; FBDGs, food-based dietary guidelines; PI/ECOTSS, population, intervention/intake/exposure, comparator, outcome, timing, setting, study design.

Figure 2 outlines how SR topics will be prioritized and selected. The following section gives a brief description of the process, followed by a more in-depth discussion.

The process starts with the formulation of an initial draft ScR for each chapter by the NNR2022 Committee. The drafts and inputs from a public call is then discussed by the NNR2022 Committee; the discussion concludes with a decision on whether each topic should be shortlisted or not (shortlisting criteria, see below). If shortlisted, the NNR2022 Committee formulates a formal research question (PI/ECOTSS statement). The NNR2022 Committee then examines whether a recent qualified SR exists on the same topic. If a qualified SR exists, the topic is not prioritized for *de novo* SR by the NNR2022 project, but the qualified SR will instead be used. If no previous relevant qualified SR exists, the PI/ECOTSS statement will be considered for prioritization. The final list of prioritized topics is then ranked by the NNR2022 Committee and consulted by the Scientific Advisory Group. If prioritized above a certain threshold (defined by available financial resources), the topic is commissioned to the NNR SR Centre.

Box 1 Scoping reviews

A scoping review (ScR) is a relatively new type of review. Like in a systemic review (SR), subjective choices made during the review process are clearly described, making it more transparent than a narrative review. In ScRs, the specific methods by which the included studies are identified, selected, and evaluated are always reported. Thus, although not as comprehensive as in an SR, the conclusions of a ScR are likely to be less colored by bias than those of a narrative review.

SRs are often used for a limited number of and narrowly defined questions, while ScRs are used for broader questions (such as all the health outcomes covered in a chapter). ScRs are also useful when the literature has not recently been comprehensively reviewed by an SR.

Shortlisting and prioritization is done according to the following criteria:

- Relevance: The topic is within the scope of NNR2022.
- Importance: The topic has new, relevant data in an area of substantial public health concern.
- Potential national impact: The SR may inform national food and health policies and programs.
- Avoiding duplication: The topic is not currently addressed through other qualified SRs (see inclusion and exclusion criteria for qualified SRs in the ‘Identification of qualified Systematic reviews’ section that can be used in the evaluation of causal association).

When a topic is selected for NNR2022 SR, the scientific research question will be formulated as a formal PI/ECOTSS statement. The PI/ECOTSS statements will consist of the following elements: population; intervention, intake or exposure; comparators; outcomes; timing; setting; and study design.

All NNR2022 SRs will strictly follow The Nordic Nutrition Recommendations 2022 – Structure and rationale of qualified systematic reviews (2) and The Nordic Nutrition Recommendations 2022 – Handbook for qualified systematic reviews (3). For an in-depth discussion of the elements of the PI/ECOTTS statement, please consult these companion papers.

## Identification of qualified SRs

Many SRs have been published on the effect of nutrients or foods on health outcomes. Some SRs are of very high quality and may be used as part of the evidence base in NNR2022. Inclusion and exclusion criteria for qualified SRs in the NNR2022 project are summarized below. To qualify, all criteria must be met.

### Inclusion criteria

- Commissioned by national food or health authorities, or international food and health organization
- Authored by a multidisciplinary group of experts
- Consists of an original SR of the evidence for a nutrient/food–health relationship
- Includes at least one nutrient/food topic, and its relationship to at least one outcome related to a chronic disease or condition that is of public health interest in Nordic or Baltic countries
- Includes a clear description of SR methodology, which should be similar to the methodology used NNR2022 (companion paper)
- Includes an assessment of the quality of primary studies
- Provides an evidence grade for the overall quality of the evidence
- English language

### Exclusion criteria

- SR should not be commissioned or sponsored by industry or an organization with a business or ideological interest
- SR should not be updated later in another qualified SR on the same topic
- SR should not focus on an outcome outside the scope of the NNR (e.g. disease management or food safety).

Qualified SRs will be included in the evidence base of each chapter together with the SRs developed as part of the NNR2022 project. A limited number of qualified SRs from national or international authorities and organizations have been publised (8). The following organizations will also be contacted for information about relevant, qualified SRs not published through traditional scientific journals:

- National Academy of Scienes, Engeneering and Medicine, the United States
- Dietary Guidelines Advisory Committee, the United States
- World Health Organization (WHO)
- World Cancer Research Fund (WCRF)
- European Food Safety Agency (EFSA)
- Scientific Advisory Committee on Nutrition (SACN), the United Kingdom
- German Nutrition Society, Germany
- Health Council, The Netherlands
- National Health and Medical Research Council, Australia
- Ministry of Health, New Zealand
- Health Canada, Canada

Other available qualified SRs will be searched for by the Committee. SRs by individual groups of scientists, even though not considered qualified in the NNR2022 project, can be useful in *other* subsections of the NNR2022 chapters (Table 1) but will not be identified and used as ‘qualified’.

## The systematic approach to develop DRVs and FBDGs

**Fig. 3:**
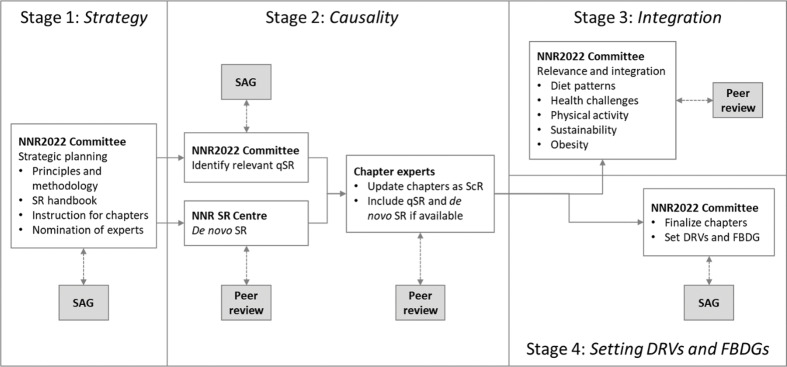
Process for defining DRVs and FBDGs: four stages. Abbreviations: SAG; Scientific Advisory Group; qSR, qualified systematic review; ScR, scoping review; NNR SR Centre, NNR Systematic Review Centre; DRVs, dietary reference values; FBDGs, food-based dietary guidelines.

A main output of the NNR2022 is to provide evidence-based DRVs and FBDGs for the Nordic and Baltic countries. The work to develop DRVs and FBDGs involves four separate stages (Fig. 3). In the following, we give a brief description, followed by an in-depth discussion of each stage.

First, the NNR2022 Committee defined an *a priori* plan or strategy for how to update DRVs and FBDGs. Second, the scientific evidence for causal associations between nutrient/food exposures and health outcomes will be reviewed. Importantly, qualified SRs will constitute a main foundation in order to establish these causal associations. The third stage will involve the evaluation of the relevance of the causal associations for the Nordic and Baltic countries, with special reference to integration of sustainability issues. The fourth and final stage will involve the final development and formulation of DRVs and FBDGs.

Historically, the DRVs defined by the NNR are implemented in each Nordic and Baltic country in its entirety, with only occasional small national adjustments. On the other hand, formulation of FBDGs also considers national context, policy, food tradition, and preferences, and has traditionally been issued nationally. The sustainability of food systems in the countries may also influence local adjustment of FBDG. Importantly, the scientific evidence base for both DRVs and FBDGs is the same for all the countries involved in the NNR2022 project.

## Stage 1. Strategic planning for how to update DRVs and FBDGs

In the first stage, the NNR2022 Committee laid out a detailed *a priori* plan of the entire process to update DRVs and FBDGs. A well-defined *a priori* plan is essential for complete transparency and to avoid biases that may arise during the process. The present paper and two companion papers (2, 3) defines the overarching guiding strategic principles, and the theoretical and practical aspects related to *de novo* SR synthesis. In addition, the NNR2022 Committee will define instructions for how topic experts will write individual NNR chapters.

## Stage 2. Evaluation of evidence for causal associations

Next, the evidence for causal associations between nutrients, food, and/or drinks and health outcomes will be evaluated (Section ‘Health outcomes relevant for Nordic and Baltic countries’ in Table 1).

Mainly three study types are used to set DRVs and FBDGs: 1) clinical trials (preferably RCTs); 2) observational studies (preferable prospective cohort studies or Mendelian randomization [MR] studies); and 3) mechanistic studies (i.e. physiology, metabolism, biochemistry, and molecular functions) (Boxes 2 and 3). SRs are the preferred method to synthesize the totality of evidence across each study type.

Well-designed and well-implemented RCTs can demonstrate causality, while observational studies, in the absence of huge effect sizes, do not. RCTs are not, however, always available (see below).

The methodology for SRs normally used within the field of clinical medicine is based on the methods described by The Cochrane Collaboration (9) and the Grading of Recommendations Assessment, Development and Evaluation (GRADE) working group (24). In such works, it is generally accepted that a hierarchical system is used for documentation, where RCTs are accorded more weight than observational studies, as RCTs are less affected by various sources of error. RCTs are appropriate in clinical medicine, since the research question often involves evaluation of the effect of an intervention, such as a pharmaceutical or surgical intervention, compared to a control group.

For ethical reasons, long-term RCTs on deficiency or toxicity are not – and *will* not – be available. When considering nutrient deficiency, adequacy, or toxicity, other types of studies are more often used: mechanistic studies, studies in animals or humans on nutrition status and metabolism, and balance studies. Prospective cohort studies may also be useful.

RCTs are not realistic for many chronic diseases that develop slowly over a long period, often several decades. It is not ethical, practical, or economically feasible to conduct RCTs with decades long lifestyle interventions. Clinical trials that randomize healthy people to a presumably unhealthy lifestyle, such as a poor diet, smoking, or physical inactivity, and then wait for decades to test whether the intervention arm dies prematurely has not, and should not, be performed. Evaluation of the evidence for causal associations between lifestyle factors, such as diet, smoking, physical activity, stress, sleep, and other lifestyle habits, must therefore apply a more holistic and sophisticated strategy, which indeed differs in important aspects from the methods used by, for example, Cochrane (9) and GRADE (24).

There are some important exceptions where RCTs are also available and useful when considering DRVs and FBDGs (see Box 2 for details).

The basis for the dietary advice for a population is linked to etiology, where an attempt is made to identify the role of food in the risk of chronic diseases. Causality in such a context must be based on an overall assessment of the documentation from various types of studies, such as clinical intervention studies (particularly RCTs with intermediate outcomes and/or relatively short-term interventions), epidemiological studies (particularly prospective observation and MR studies), and physiological and mechanistic studies (particularly basic experimental research using cell- and animal-based models). The sum of this literature forms a basis for assessments of causality in accordance with the methodology described by the WCRF (14, 15), the WHO (16), Bradford-Hill (17, 18), Rothman and Greenland, and others (19, 20). The methodology behind the dietary recommendations is therefore inclusive and draws on all types of relevant knowledge to a considerable extent, unlike more hierarchical systems that are used to assess the effects of pharmaceuticals and other interventions.

## Stage 3: Integration with other aspects relevant for the Nordic context

The third stage involves the evaluation of the *relevance* of the causal associations for the Nordic countries. This stage takes into account the characteristics of the Nordic and Baltic populations, including the current patterns of dietary consumption, and diet-related health challenges in the Nordic and Baltic countries. The NNR will also have a special emphasis on integration of aspects related to physical activity, obesity, and sustainability. The implications of the boundaries defined by the 1) Nordic and Baltic diet pattern, 2) Nordic and Baltic health challenges, 3) physical activity, 4) obesity, and 5) sustainability will be described in separate chapters.

Box 2 Various types of scientific study are necessary in order to assess causality

Each individual study type, all with strengths and weaknesses, cannot alone form the basis for drawing causal conclusions and setting dietary reference values and food-based dietary guidelines. Collectively, they can however provide an adequate basis for dietary advice. Some of the strengths and weaknesses of each study type are described below.

***Intervention studies***

Among intervention studies, randomized controlled trials (RCTs) give the best evidence of causal associations, since this type of study can provide the best control for confounding factors (10, 11).The advantage of intervention studies is that the effects of the intervention are studied in people under (partly) controlled conditions, but the disadvantage is most often the short duration of exposure or study periods. Lifestyle habits such as diet and physical activity are difficult to control over a long period of time in free-living people, but their impact on health develops over a long period of time. In addition, adequate controls for foods and lifestyle habits are difficult to achieve and poor compliance with the intervention complicates the interpretation of the results. Such studies therefore normally only cover a small part of the development of the disease, and often lack post-trial follow-up on the long-term effects of the intervention. A notable exception are studies focusing on type 2-diabetes.

For a few diseases, surrogate disease markers can be used as substitutes for chronic disease outcomes. Examples of such qualified surrogate markers are low-density lipoprotein -cholesterol, serum total cholesterol, and blood pressure for cardiovascular diseases; bone mineral density for osteoporosis; adenomatous polyps for colorectal cancer; and increased blood glucose and insulin resistance for type 2-diabetes. In such cases, RCTs may be more feasible in evaluating causality.

***Observational epidemiological studies***

Strengths of longitudinal epidemiological studies include a long observation period and that morbidity and mortality can be included as endpoints. Effects of foods in free-living people without any restrictions can thus be studied. It is however difficult to control for all confounding factors. In addition, adequate methods for assessing diet in sufficient detail in epidemiological studies are generally not available. Epidemiological studies also require differences in exposure levels between individuals in the population groups being studied, which is often not the case within a population. External validity of observational studies is largely related to representativeness of the cohort (e.g. geographic location, stability of the cohort on follow-up).

Prospective cohort studies have the fewest sources of error, and the results of such studies therefore provide the highest level of evidence among epidemiological types of study (10–12). Observational studies cannot by themselves prove or falsify causal associations, or existing knowledge. One exception is Mendelian Randomization studies, explained in detail in Box 3.

***Mechanistic in vitro studies, studies involving cell cultures and experimental animals***

These studies are important in order to identify biological mechanisms. Such studies can be carried out under highly controlled conditions, and there is considerable scope to use many different exposures (doses, time, individual substances, etc.). However, it can be difficult to transfer results from *in vitro* and cell tests to a physiological context, and it is challenging to transfer results from controlled animal experiments to free-living people. Tests using ‘knockout’ mice and other transgenic mice are particularly important for establishing biological mechanisms in a physiological context. The revolution in molecular biology and the sequencing of the human genome have provided ground-breaking new insights into mechanisms and our biological understanding of nutrients, other bioactive substances in diet, and the development of diseases. In addition, the long-term regulation of genes in connection with epigenetic mechanisms can be an important factor in explaining how lifestyle habits can affect the risk of disease, a risk which can also be passed on to the next generation (13).

***Various types of scientific study are necessary in order to assess causality***

Together, these three types of study will form the basis for assessments of causality in accordance with the methodology described by the World Cancer Research Fund (WCRF) (14, 15), the World Health Organization (WHO) (16), Bradford-Hill (17, 18), Rothman and Greenland and others (19, 20).

## Stage 4: Formulation of DRVs and FBDGs

The fourth stage involves the final development and formulation of DRVs and FBDGs. While the scientific evidence for causal associations and the Nordic and Baltic context are the main bases for the specific DRVs and FBDGs, several other aspects will also be taken into account.

The evidence base for DRVs and FBDGs may be different. For example, the level of certainty of a causal relation between exposure and outcome may often be quite different when assessing nutrient deficiency, nutrient adequacy, upper limits of nutrients, and optimal intake of food groups. In spite of these differences, we aim at, whenever possible, applying similar principles and methodologies when setting the two kinds of recommendations.

SRs will likely find that certain aspects within each chapter (nutrient, food, or drink) are unknown or uncertain. There may be lack of data, the studies may be very old, studies may have a high risk of bias, or there may be uncertainties related to biomarkers or methods for extrapolation to certain age groups. Still, for many nutrients, we will formulate DRVs in spite of uncertainties in these assessments. In the final NNR report, we will describe transparently all the relevant uncertainties for each nutrient, food group. Thus, a holistic assessment will lie behind the decisions to formulate FBDGs.

Box 3 Mendelian randomization studies

Epidemiological studies cannot infer causality due to two major limitations: confounding and reverse causality (21).

Mendelian randomization (MR) studies, on the other hand, combine the strengths from both epidemiological studies and randomized controlled trials (RCTs). Similar to an epidemiological study, a MR study is observational – it aims to quantify the association between an exposure (a biomarker) and a disease outcome. In addition, similar to an RCT, an MR study is also interventional. The basis for these unique features of the MR methodology is the random segregation of gene variants, or alleles (usually caused by single nucleotide polymorphisms, SNPs), during meiosis. Consequently, confounding factors should be equally distributed between subjects with different alleles. In addition, since the segregation of alleles precedes the development of disease outcomes, MR studies should be free from reverse causation (21).

**Fig. 4:**
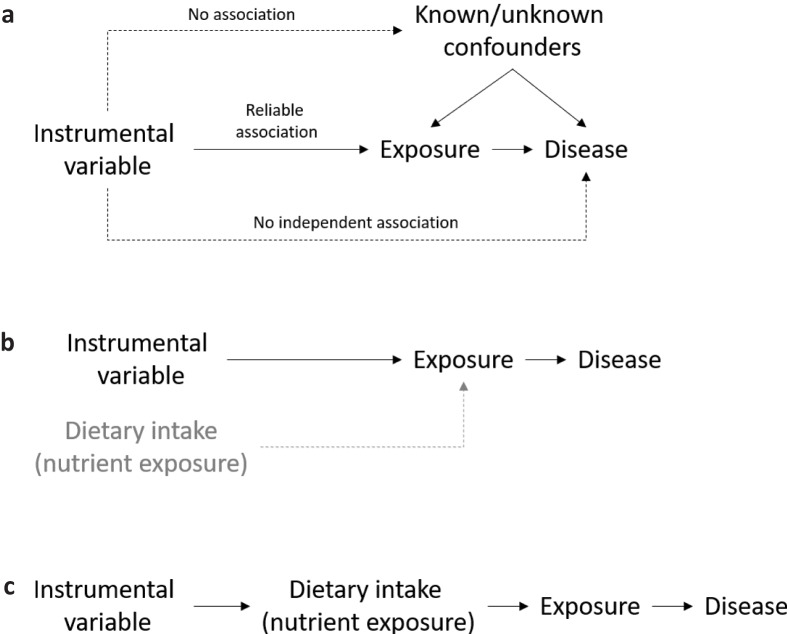
Principles of Mendelian randomization in nutrition research. (a) Instrumental variable analysis. Figure adapted from (21). (b) Dietary interventions that affect causal disease exposures are clinically important. (c) Instrumental variables can sometimes be used as proxies for dietary intake.

MR studies can be particularly relevant for situations where specific alleles directly affect the level or function of gene product. If the underlying molecular relationships between gene variants, protein level and function, and physiology are thoroughly described, the allele can constitute a so-called instrumental variable for a specific exposure or biomarker. The three fundamental assumptions underlying this MR concept is that 1) genes are randomly assigned among people, 2) some genes influence the exposure of interest, and 3) the genes that influence the exposure do not influence the outcome via any other path than via the exposure (22) (Fig. 4a).

Documentation of causality for certain risk factors (low-density lipoprotein cholesterol, triglycerides, blood pressure) and non-causality for others (high-density lipoprotein cholesterol), have implications for the strength of evidence of nutrition recommendations. Importantly, in light of MR studies, dietary interventions that report sufficiently large changes in causal risk factors can be considered highly clinically relevant: although they show changes in intermediate endpoints, we may now more precisely extrapolate to hard endpoints, such as coronary heart disease (CHD) morbidity and mortality (Fig. 4b). In this respect, some MR studies may mimic lifelong exposure to certain dietary habits and lifestyles, their associated risk profiles, and disease outcomes.

In some MR studies, instrumental variables may be used to objectively assess intake or exposure to certain nutrients (Fig. 4c). For example, alleles in the gene ALDH2 associate with varying degrees of flushing upon alcohol exposure, and across the three potential bi-allelic combinations (major-allele homozygotes, heterozygotes, and minor-allele homozygotes), subjects consequently exhibit lower intake of alcohol. Using this allele as an instrumental variable for alcohol exposure, researchers have shown that alcohol linearly increases blood pressure and the risk of CHD, with no lower threshold (21).

In recent years, gene-exposure-outcome associations examined using MR studies have become more and more complex, including those within nutrition research, which makes it harder to robustly conduct and interpret the findings (21). Because MR studies can suffer from many of the same biases as other study types, they must be subjected to critical evaluation in SR syntheses. For example, conventional inverse variance-weighted MR analysis can be evaluated in light of an MR-Egger analysis that also evaluates biases caused by horizontal pleiotropy (21). VanderWeele and co-workers provided an in-depth discussion on similar methodological challenges in MR studies, including settings with inadequate phenotype definition, the setting of time-varying exposures, the presence of gene–environment interaction, the existence of measurement error, the possibility of reverse causation, and the presence of linkage disequilibrium (23).

In summary, MR studies can be considered observational experiments of nature, and well-conducted MR studies can determine causality of associations between exposures and disease outcomes (23). Some MR studies are relevant for nutrition research and dietary recommendations but interpretation and implementation require thorough evaluation similar to other study types.

Food-based scientific studies will be used as a starting point for setting FBDGs; however, if food groups are of particular importance for certain nutrients, nutrient-based research will also be included. Formulating FBDGs must also take into consideration the entirety of the diet, Nordic and Baltic food traditions, and developments in the Nordic and Baltic diets. The FBDGs set in the NNR will be general, mainly on food group level, allowing each country to develop more specific national FBDGs, if required.

For FBDGs, we will consider to include nutrient considerations for each food category if 15% of a nutrient originates from that food category. This is in line with the European regulations on the declaration of nutrients in foods (No 1169/2011): a quantity or source is defined as being ‘significant’ if the food contains 15% or more of the DRVs.

### Setting dietary reference values

For chapters in NNR that concern nutrients, qualified SRs will inform the NNR2022 Committee on the scientific evidence that is relevant for setting DRVs. In the following, we give a general description of the considerations and the principles for setting DRVs.

The DRVs are intended for healthy individuals. Generally, the DRVs cover increased requirements such as during short-term mild infections or certain medical treatments. The DRVs are usually not suited for long-term infections, malabsorption, and various metabolic disturbances or for treatment of persons with a suboptimal nutritional status. They are meant to be used for prevention purposes. The NNR, however, does cover dietary approaches for sustainable weight maintenance after significant, intentional weight reduction. For individuals with disease and for other groups with special needs, the dietary composition might have to be adjusted accordingly.

Where original data are lacking or insufficient, extrapolation from one group to another is often necessary. The most common method is to extrapolate values from adults to children using a weight or metabolic factor and adjusting for growth. This approach will also be applied in the current NNR.

**Fig. 5:**
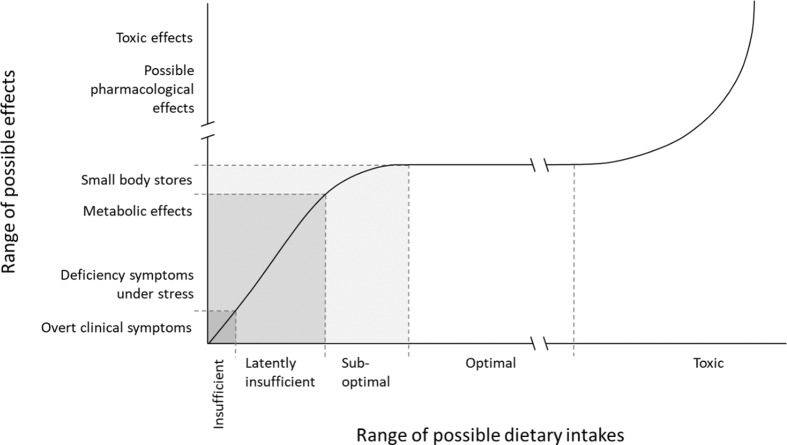
The theoretical relationship between intake of a nutrient and the effect on the organism.

For most nutrients, a hierarchy of criteria for nutrient adequacy can be established ranging from prevention of clinical deficiency to optimal levels of body stores and functionality (Fig. 5). A higher intake of a nutrient is, however, not necessarily better for health. Beyond a certain intake level, a higher intake might even lead to adverse health effects. Therefore, it is common to talk about a safe intake window, where intakes lower or higher than indicated by the window might affect health negatively.

### Average requirement

**Fig. 6:**
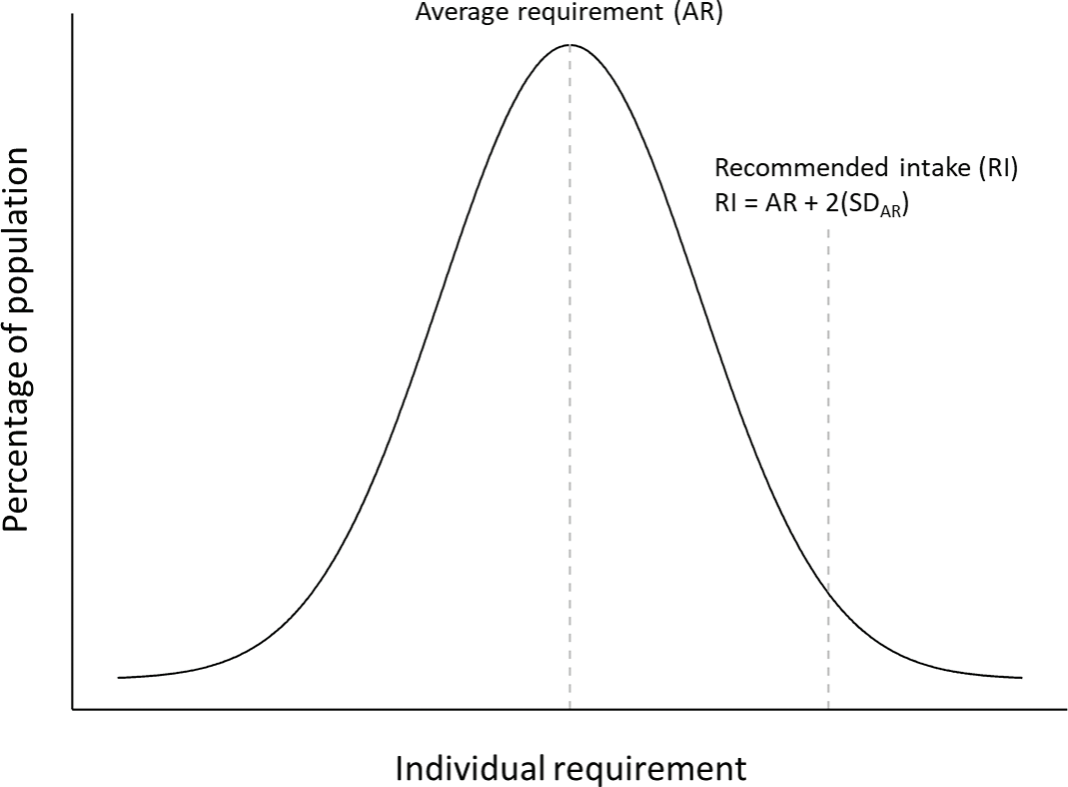
Frequency distribution of an individual nutrient requirement. Abbreviations: SD, standard deviation.

In NNR, the AR is defined as the value to meet the requirement for half of a defined group of healthy individuals provided that there is a normal distribution of the requirement (Fig. 6).

In general, the selected criteria for establishing the AR apply to micronutrients and are usually based on data on biochemical markers of adequate nutritional status. However, the AR can also be derived for some macronutrients such as protein and essential fatty acids.

Deficiency of a nutrient would imply that the supply is so small that specific symptoms of disturbances in body functions emerge. During serious, manifest deficiency, overt clinical symptoms or signs such as bleeding of the gums during scurvy or neurological symptoms due to vitamin B12 deficiency would arise. Data on biochemical markers can include the activity of certain enzymatic systems in which nutrients have a role as cofactors or concentrations of a nutrient in cells or fluids as a measure of tissue stores. Low activities or concentrations might be associated with deficiency symptoms or impaired function. Moreover, it is possible to define an interval between manifest deficiency and optimal intake level in which clinical symptoms are more diffuse or do not exist at all. This level is sometimes called latently insufficient (Fig. 5). Such indicators are available only for a limited number of nutrients, for example, for vitamin D, iron, folate, and vitamin B12.

The definition of *AR* corresponds to the term ‘Estimated Average Requirement’ (EAR) used in the UK and US recommendations (25, 26). The EFSA uses the term ‘Average Requirement’ (27).

It is important to distinguish between the AR for a nutrient and the RI of a nutrient. The RI represents more than the requirement for the average person and also covers the individual variations in the requirement for the vast majority of the population group (Fig. 6). Depending on the criteria used for setting the AR, the safety margin between the AR and RI can vary.

### Recommended intake

RI refers to the amount of a nutrient that meets the known requirement and maintains good nutritional status among practically all healthy individuals in a particular life stage or gender group. When the distribution of a requirement among individuals in a group can be assumed to be approximately normally distributed (or symmetrical) and a standard deviation (SD) can be determined, the *RI* can be set as follows (Fig. 6): RI = AR + 2(SD_AR_).

For nutrients where data about the variability in requirements are insufficient to calculate an SD_AR_, an approximate coefficient of variation (CV) of 10–15% can be used (Fig. 6).

The *RI* corresponds to the amount of a nutrient that is consumed, and this means that losses during handling, preparation, processing, etc., have to be taken into consideration in dietary planning. The *RI* is appropriate for an average intake of a group expressed per day over a longer period of 1 week or more. The body can adapt and retain some nutrients when the intake is lower than the immediate requirement. The storage capacity for nutrients varies and is highest for the fat-soluble vitamins (several months) while the stores of water-soluble vitamins (with the exception of vitamin B12) are usually lower.

Where sufficient scientific evidence is available on interactions with other dietary factors, these are accounted for. Examples are the enhancing effect of ascorbic acid on non-heme iron absorption and the effect of folate on homocysteine levels in the blood. When establishing the RI values, these aspects have been taken into consideration.

High doses of certain vitamins and minerals can have pharmacological effects different from their primary nutritional effects. Generally, this concerns amounts that the target group could not normally obtain from the diet. The effect of high doses of nicotinic acid as a lipid-lowering agent and the effect of fluoride on dental caries can be considered pharmacological rather than nutritional effects. Such effects have not been taken into consideration in the establishment of the RI (Box 4).

The RI is intended for healthy individuals and is not necessarily appropriate for those with different needs due to diseases such as infections. In general, the RIs are only applicable when the supply of other nutrients and energy is adequate.

The definition of RI corresponds to the term ‘Recommended Intake’ used in the United Kingdom and ‘Recommended Dietary Allowance’ (RDA) used in the United States (25, 26). The EFSA uses the term ‘Population Reference Intake’ (PRI) to denote ‘the level of nutrient intake that is enough for virtually all healthy people in a group’ (27).

In some cases, data are not available to establish AR or RI. In such cases, we will consider establishing Adequate Intake. This will be discussed in forthcoming publications.

### Upper intake level

**Fig. 7:**
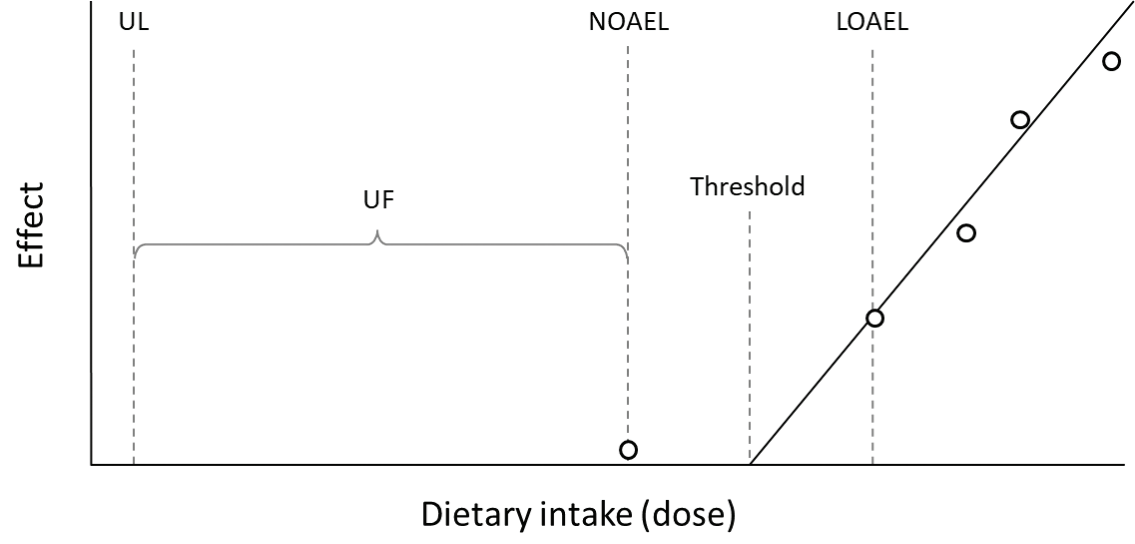
Derivation of Upper Intake Level. Abbreviations: UL, upper intake level; UF, uncertainty factor; NOAEL, no observed adverse effects level; LOAEL, lowest observed adverse effects level.

For most nutrients, high intakes might cause adverse effects or even toxic symptoms. The UL is defined as the maximum level of long-term (months or years) daily nutrient intake that is unlikely to pose a risk of adverse health effects in humans. The threshold for any given adverse effect varies depending on life-stage, sex, and other individual characteristics just as it does for any nutrient requirement. However, there are insufficient human data to establish distributions of thresholds for each adverse effect. The different steps in setting the UL include the identification of the critical endpoint, which is the lowest dose at which an adverse effect occurs, and using a surrogate measure for the threshold (Fig. 7).

Box 4 Setting recommended intake for micronutrients

In setting recommendations for micronutrients, the Nordic Nutrition Recommendations use the classical approach with the following steps:

The first step includes an evaluation of the average physiological and dietary requirement for the population group in question as judged by criteria that have to be set specifically for every individual nutrient. The establishment of these criteria includes considerations of clinical and biochemical deficiency symptoms, body stores, body pool turnover, and tissue levels. The nutritional requirements are influenced mainly by different biological factors such as age, sex, growth, height, weight, pregnancy, and lactation.

The second step includes an estimation of a safety margin to ensure that all individual variations are considered and added to the requirement to obtain a level of recommended intake. The size of this safety margin depends on several factors, among others the variation in the requirements between individuals and potential adverse effects of high intakes. Furthermore, the precision of the estimation of the requirement should be taken into consideration (Fig. 6).

The thresholds are the following:

- No observed adverse effect level (NOAEL): the highest intake of a nutrient with no observed adverse effects
- Lowest observed adverse effect level (LOAEL): the lowest intake level with an observed adverse effect.

Based on these evaluations, a UL is derived by taking into account the scientific uncertainties in the data by dividing the NOAEL by an uncertainty factor (UF) (Fig. 7). This factor should account for uncertainties in human inter-variability or, in the case of insufficient human data, an extrapolation from animals to humans as well as other uncertainties or deficiencies in the data. The definition of UL corresponds to the term ‘Tolerable upper intake level’ used in the United States (25) and by the EFSA (27).

### Lower intake level

The LI is defined as a cutoff intake value below which an intake could lead to clinical deficiency symptoms in most individuals. Establishment of an LI is thus based on observations of individuals and is in many cases based on criteria other than the AR.

The definition of *LI* differs from the term ‘Lower reference nutrient intake’ (LRNI) used in the United Kingdom, which is defined as EAR minus 2 SD (26). The EFSA uses the term ‘Lower threshold intake’ (LTI) to define the level of intake below which almost all individuals will be unlikely to maintain ‘metabolic integrity’ according to the criterion chosen for each nutrient (27).

### Reference values for energy intake

The term *reference value for energy intake* refers to the calculated estimated energy requirement for groups of healthy individuals with normal body size and various levels of physical activity. Setting the reference value for energy intake requires a different approach, compared to the reference values for vitamins and minerals. For some vitamins and minerals, RIs can be given with large margins because the absorption can be limited, or the excess can be broken down or secreted. The RIs might therefore exceed the defined requirements of the individual on a long-term basis. For energy intake, the situation is different because an energy intake consistently above or below the energy requirement will result in weight gain or weight loss that can adversely affect health. Consequently, and to prevent under- or overconsumption, energy intake should equal energy expenditure. The reference value for energy intake is expressed as the average energy requirement for a defined population group with various levels of physical activity (excluding competitive athletes). Thus, the reference value for energy intake should be considered a theoretical value intended to be used as a reference for the entire population group.

### Recommended intake range of macronutrients

The term *RI range of macronutrients* (i.e. percentage of energy from macronutrients) is used to emphasize the importance of the distribution of energy between energy-providing nutrients. The current major lifestyle diseases mainly result from overnutrition and nutritional imbalances rather than from under-nutrition and deficiency symptoms. The intention of setting the RI range of macronutrients is, therefore, to derive a dietary macronutrient composition that will provide an adequate intake of essential nutrients for optimal health and a reduced risk of major lifestyle diseases (Fig. 6).

The RI range of macronutrients is based on an overall assessment of current knowledge about the impact of macronutrient intake on health and/or risk of disease. This requires various types of scientific data primarily from RCTs, prospective cohort studies, and other epidemiological studies. Where possible, studies providing evidence of a causal relationship and dose–response effects are shown. A direct causal relationship between the intake of a single nutritional factor and a specific function or selected criterion, such as reduction of the risk of diseases, is not always evident from the scientific data due, for example, to interactions between several energy-providing nutrients. In such cases, effects due to substituting different energy-providing nutrients under energy-balance conditions are taken into consideration (e.g. replacing saturated fat with unsaturated fat or complex carbohydrates). In these cases, the RI range of macronutrients is based on an overall assessment of the scientific evidence and includes specific considerations about known patterns of intake of nutrients and foods, and the actual composition of available foods in the Nordic countries. On this basis, the RI range of macronutrients should be considered ‘optimal’ for the Nordic and Baltic conditions.

The RI range of macronutrients refers to appropriate ranges of the usual intake in the majority of individuals in the population (7). For planning purposes, a value approximately in the middle of this range can be used as the target. For certain macronutrients, *upper* and *lower* thresholds are defined. These thresholds refer to maximum and minimum levels of dietary intake for which a population is recommended to adhere; for example, for saturated fat and added sugar (upper thresholds), and dietary fiber (lower threshold).

## Summary and conclusions

In this paper, we have described the organization, principles, methods, and the systematic approach that will be used in the development of NNR2022. The paper has defined *a priori* all methodology and data handling for clarity and transparency. This paper is the first of three and should be viewed in concert with its two companion papers, namely, *Structure and rationale of qualified systematic reviews* (2), which describes all aspects related to the SR methodology, and *Handbook for qualified systematic reviews* (3), which describes the NNR2022 project plan to conduct SRs according to the structure and rationale outlined in the companion paper.

We believe and hope that these principles and methods also can serve as a state-of-the-art basis for other national food and health authorities that plan to develop DRVs and FBDGs. See section on “Conflicts of interest” and “Sponsors of the NNR2022 project”

## References

[1] Nordic Council of Ministers Nordic Nutrition Recommendations 2012: integrating nutrition and physical activity. Nordic Nutrition Recommendations. Report No: 5. Copenhagen: Nordic Council of Ministers; 2014, p. 627. doi: 10.6027/Nord2014-002

[2] Arnesen EK, Christensen JJ, Andersen R, Eneroth H, Erkkola M, Høyer A, et al. The Nordic Nutrition Recommendations 2022 – Structure and rationale of qualified systematic reviews. Food Nutr Res 2020. doi: 10.29219/fnr.v64.4403 PMC7307429 32612488

[3] Arnesen EK, Christensen JJ, Andersen R, Eneroth H, Erkkola M, Høyer A, et al. The Nordic Nutrition Recommendations 2022 – Handbook for qualified systematic reviews. Food Nutr Res 2020. doi: 10.29219/fnr.v63.4404 PMC7307435 32612492

[4] Mozaffarian D Conflict of interest and the role of the food industry in nutrition research. JAMA 2017; 317(17): 1755–6. doi: 10.1001/jama.2017.3456 28464165

[5] Soares MJ, Müller MJ, Boeing H, Maffeis C, Misra A, Muscogiuri G, et al. Conflict of interest in nutrition research: an editorial perspective. Eur J Clin Nutr 2019; 73(9): 1213–5. doi: 10.1038/s41430-019-0488-8 31485036

[6] Liberati A, Altman DG, Tetzlaff J, Mulrow C, Gøtzsche PC, Ioannidis JPA, et al. The PRISMA statement for reporting systematic reviews and meta-analyses of studies that evaluate health care interventions: explanation and elaboration. PLoS Med 2009 21; 6(7): e1000100. doi: 10.1371/journal.pmed.1000100 19621070 PMC2707010

[7] Tricco AC, Lillie E, Zarin W, O’Brien KK, Colquhoun H, Levac D, et al. PRISMA Extension for Scoping Reviews (PRISMA-ScR): checklist and explanation. Ann Intern Med 2018; 169(7): 467–73. doi: 10.7326/M18-0850 30178033

[8] Blake P, Durão S, Naude CE, Bero L An analysis of methods used to synthesize evidence and grade recommendations in food-based dietary guidelines. Nutr Rev 2018; 76(4): 290–300. doi: 10.1093/nutrit/nux074 29425371 PMC5914460

[9] Higgins JP, Green S (editors). Cochrane handbook for systematic reviews of interventions version [Internet]. The Cochrane Collaboration, 2019. Report No.: 6. 2019. Available from: handbook.cochrane.org [cited 2019 November 24].

[10] Laake P Epidemiologiske og kliniske forskningsmetoder. Oslo: Gyldendal Akademisk; 2007.

[11] Aalen OO, Frigessi A Statistiske metoder i medisin og helsefag. Oslo: Gyldendal Akademisk; 2008.

[12] Thelle D Innføring i epidemiologi. Oslo: Cappelen Akademiske Forlag; 2004.

[13] Alberts B Molecular biology of the cell: reference edition. New York: Garland Science; 2008.

[14] World Cancer Research Fund/American Institute of Cancer Research Continuous update project expert report – the third expert report; 2018. Diet, nutrition, physical activity and cancer: a global perspective. Available from: dietandcancerreport.org [cited 2019 November 24].

[15] World Cancer Research Fund/American Institute of Cancer Research Judging the evidence [Internet]. Continuous update project expert report – the third expert report; 2018. Diet, nutrition, physical activity and cancer: a global perspective. Available from: dietandcancerreport.org [cited 2019 November 24].

[16] World Health Organization (WHO) Diet, nutrition and the prevention of chronic diseases: report of a joint WHO/FAO expert consultation. Uncertain: WHO Technical Report Series 2003.

[17] Hill AB The environment and disease: association or causation? J R Soc Med 2015; 108(1): 32–7. doi: 10.1177/0141076814562718 25572993 PMC4291332

[18] Hill AB, Hill ID Bradford Hills’s principles of medical statistics. London: Edward Arnold, 1991.

[19] Rothman KJ, Greenland S, Lash TL Modern epidemiology. Philadelphia: Wolters Kluwer/Lippincott Williams & Wilkins; 2008.

[20] Maldonado G, Greenland S Estimating causal effects. Int J Epidemiol 2002; 31(2): 422–9. 11980807

[21] Holmes MV, Ala-Korpela M, Smith GD Mendelian randomization in cardiometabolic disease: challenges in evaluating causality. Nat Rev Cardiol 2017; 14(10): 577–90. doi: 10.1038/nrcardio.2017.78 28569269 PMC5600813

[22] Koellinger PD, de Vlaming R Mendelian randomization: the challenge of unobserved environmental confounds. Int J Epidemiol 2019; 48(3): 665–71. doi: 10.1093/ije/dyz138 31263889 PMC6659461

[23] VanderWeele TJ, Tchetgen Tchetgen EJ, Cornelis M, Kraft P Methodological challenges in Mendelian randomization. Epidemiology 2014; 25(3): 427–35. doi: 10.1097/EDE.0000000000000081 24681576 PMC3981897

[24] Schünemann H, Brożek J, Guyatt G, Oxman A (editors) GRADE handbook for grading quality of evidence and strength of recommendations [Internet]. Report No.: 6. Cochrane methods: The GRADE Working Group; 2013 [cited 24 November 2019]. Available from: gdt.guidelinedevelopment.org/app/handbook/handbook.html

[25] Otten JJ, Hellwig JP, Meyers LD (editors). Dietary reference intakes: the essential guide to nutrient requirements. Washington, DC: Institute of Medicine (IoM) of the National Academies; 2006.

[26] Dietary reference values for food energy and nutrients for the United Kingdom Report of the panel on dietary reference values of the Committee on Medical Aspects of Food Policy. Rep Health Soc Subj (Lond). 1991; 41: 1–210. 1961974

[27] EFSA Panel on Dietetic Products, Nutrition, and Allergies (NDA) Scientific opinion on principles for deriving and applying dietary reference values. EFSA J 2010; 8(3): 1458.

